# Exploring the multifaceted roles of beta-defensins in prediabetes: a detailed review

**DOI:** 10.3389/fendo.2026.1743850

**Published:** 2026-02-25

**Authors:** Nourah Almansour, Amal Hasan, Shaima Albeloushi, Rasheed Ahmad

**Affiliations:** 1 Immunology and Microbiology Department, Dasman Diabetes Institute, Dasman, Kuwait; 2 Translational Research Department, Dasman Diabetes Institute, Dasman, Kuwait

**Keywords:** diabetes progression, human beta-defensins, innate immunity, insulin resistance, metabolic inflammation, prediabetes

## Abstract

Prediabetes represents a critical window of immune–metabolic dysregulation during which insulin resistance, low-grade inflammation, and barrier dysfunction emerge before overt diabetes. Human β-defensins (HBDs), classically described as antimicrobial peptides, are increasingly recognized as modulators of epithelial integrity, inflammatory signaling, and host–microbiota interactions process central to early metabolic deterioration. Evidence from genetic, experimental, and clinical studies indicates that alterations in HBD expression accompany insulin resistance, β-cell stress, and gut barrier impairment, with tissue-specific patterns observed across the dysglycemic spectrum. While most human data derive from established diabetes, animal models and limited human observations suggest that defensin dysregulation may arise earlier and contribute to the transition from prediabetes to diabetes. Importantly, HBDs are detectable in saliva, serum, and tissues, supporting their feasibility as accessible biomarkers of early immune–metabolic stress. However, heterogeneity in assay platforms, sample matrices, and study design currently limit clinical translation. This review synthesizes current evidence linking β-defensins to early metabolic dysfunction, distinguishes associative human findings from mechanistic experimental data, and highlights critical gaps in prediabetes-focused research. We propose that β-defensins represent promising early immune–metabolic indicators whose validation in longitudinal prediabetes cohorts may improve risk stratification and enable earlier intervention.

## 1 Introduction

Diabetes mellitus (DM) and its precursor, pre-diabetes, are emerging as major global health challenges, contributing to a significant burden of illness and death worldwide. In 2021, there were 529 million individuals living with diabetes, and estimates suggest that this figure could rise to over 1.31 billion by 2050 (1). Both pre-diabetes and diabetes are characterized by metabolic imbalances, chronic low-grade inflammation (2), and an increased vulnerability to infections (3). This growing recognition of immune–metabolic interactions underscore the need to examine molecules, such as human β-defensins, that may contribute to these early physiological shifts.

Human beta defensins (HBDs), which are small cationic antimicrobial peptides produced by epithelial and immune cells, are well-known for their antimicrobial and immunomodulatory functions (4). However, emerging evidence indicates that HBDs may also play a role in metabolic regulation, insulin resistance, and inflammatory pathways associated with diabetes (5, 6). Recent human studies showed that β-defensins are clinically relevant; pancreatic biopsies from adults with type 1 diabetes had reduced expression of central innate defense molecules regardless of local inflammation, while infection-free individuals with obesity displayed higher circulating HBD-2 linked to systemic inflammation (7).

Lately, gut microbiota has gained recognition as a key contributor to early metabolic disturbances, including the transition from normoglycemia to prediabetes. Because defensins are deeply involved in maintaining gut barrier integrity and modulating immune–microbial interactions (8–10), an important scientific question arises: what roles might human β-defensins play in the progression from prediabetes to diabetes? This review brings together current genetic, clinical, and experimental evidence to explore this question and to clarify whether changes in defensin biology may serve as early indicators or contributors to metabolic deterioration. This review is structured to examine how alterations in human β-defensin biology may emerge during prediabetes and shape immune–metabolic dysfunction before the onset of overt diabetes.

This review aims to (i) synthesize genetic, experimental, and clinical evidence linking β-defensins to early immune–metabolic dysfunction, (ii) distinguish associative human data from causative animal findings, and (iii) identify critical gaps in prediabetes-focused research to guide future longitudinal and mechanistic studies.

## 2 Genetic variation evidence of HBD roles in diabetes and beyond

Although most genetic data are derived from diabetes cohorts, these findings may provide insight into immune-modifying mechanisms that operate earlier, during the prediabetic stage (11). Human β-defensins (HBDs) are small antimicrobial peptides produced by epithelial and immune cells, best known for regulating barrier integrity, innate immunity, and inflammatory signaling (12). Beyond their antimicrobial role, HBDs are increasingly recognized as modulators of pancreatic physiology (4, 13), immunometabolism pathways, and chronic low-grade inflammation (14). Variations in HBD genes, including single-nucleotide polymorphisms (SNPs) and copy number variations (CNVs), have been associated with alterations in gene expression and overall function. Although β-defensin copy number variation has been examined in cohorts with type 2 diabetes and diabetic nephropathy, current evidence shows no significant association between DEFB CNV and metabolic disease outcomes (15). Notably, early genomic studies have shown that β-defensin genes also exhibit considerable copy number variation, which influences defensin expression and innate immune responsiveness without any direct connection to metabolic disease (16). Moreover, several reported associations between β-defensin copy number polymorphisms and complex diseases have failed replication in independent cohorts, highlighting the methodological challenges and uncertain relevance of CNVs to metabolic disorders (17).

Several DEFB1 promoter polymorphisms, including rs11362, rs1799946 (18), and rs1800972 (19), have been shown to alter transcriptional activity, resulting in measurable differences in HBD-1 expression across epithelial and mucosal tissues (20). Although direct evidence that these variants modify defensin peptide structure or antimicrobial potency remains limited, diabetes-related metabolic stressors such as high glucose and dicarbonyl compounds can suppress HBD-2 (21) and HBD-3 (22) activity, suggesting that genotype and metabolic environment may interact to influence defensin function. Functionally, defensin-related polymorphisms have been associated with heightened mucosal inflammation, altered susceptibility to chronic infection, and amplification of cytokine pathways, including IL-6, TNF-α, and NF-κB (23, 24), providing a mechanistic link between defensin genetic variation, immune dysregulation, and the metabolic disturbances that underlie insulin resistance and diabetes progression.

These genetic variations can influence a person’s susceptibility to chronic inflammatory diseases, like diabetes. Polymorphic variants in the DEFB1 gene have been linked to the dysregulation of immune system function, contributing to persistent inflammatory responses that are mechanistically associated with insulin resistance and beta-cell dysfunction. Recent research reveals that individuals exhibiting comorbidity of type 2 diabetes mellitus (T2DM) and periodontitis display a heightened allelic frequency of the DEFB1 gene polymorphisms rs11362 (−20 G>A) and rs1799946. These single-nucleotide variants are significantly overrepresented in affected cohorts and correlate with altered expression profiles of DEFB1, implicating potential regulatory effects at the transcriptional level. This underscores their significance in both local and systemic immune responses (18). Among patients with type 2 diabetes, rs11362 GA and rs1799946 AA genotypes have been notably associated with the progression of diabetic kidney disease. This suggests that defensin-related pathways play a role in the complications caused by diabetes (25). In addition, a study by Martínez-Ríos et al. reported that the −44 C/G (rs1800972) polymorphism in the DEFB1 gene was significantly associated with an increased risk of developing type 2 diabetes mellitus, further strengthening the evidence that β-defensin 1 variants contribute to metabolic disease susceptibility (19). However, these findings are primarily based on relatively small, single-population cohorts, which may limit generalizability. Without replication in multi-ethnic and larger prospective cohorts, the strength of the genetic association remains undefined. There’s also evidence that rs11362 plays a role in oral mucosal immune activity, affecting HBD-1 protein levels and susceptibility to chronic inflammatory conditions (20). A limited number of investigations have explored genetic correlations within diabetic cohorts, resulting in a significant gap in current research. Expanded genetic association studies are necessary to elucidate whether HBD variants confer increased susceptibility to pre-diabetes or contribute to the acceleration of diabetes pathogenesis. These genetic insights raise essential questions about how defensin variants might influence measurable protein levels and biomarker development, which is the focus of the next section.

Importantly, large genome-wide association studies (GWAS) of type 2 diabetes have consistently identified classical metabolic susceptibility loci, most prominently TCF7L2, PPARG, SLC30A8, KCNJ11, and HHEX, that regulate β-cell function, insulin secretion, and glucose homeostasis (26–28), whereas loci encoding antimicrobial peptides such as β-defensins (including DEFB1) have not emerged as genome-wide significant drivers of disease risk. This genetic architecture suggests that DEFB1 polymorphisms are unlikely to act as primary causal genes for diabetes (26) but rather may function as modifiers of epithelial barrier integrity, mucosal immunity, and inflammatory tone within metabolic tissues, thereby shaping the tissue microenvironment in which metabolic dysfunction evolves (29). At the same time, remarkably little is known about the epigenetic regulation of DEFB1 under hyperglycemic or insulin-resistant conditions, including promoter methylation, histone modifications, or chromatin accessibility, and no transcriptomic or proteomic datasets have systematically mapped β-defensin expression across the pre-diabetes to diabetes continuum. The absence of integrated genetic, epigenetic, and multi-omics analyses therefore represents a critical gap that limits interpretation of existing association data and underscores the need for longitudinal, systems-level approaches to clarify the role of defensins in metabolic disease progression. Taken together, DEFB1 variants are unlikely to represent primary diabetogenic drivers but may act as modifiers of epithelial immunity and inflammatory tone that influence susceptibility during prediabetes (18).

## 3 Detection of HBDs: ELISA approaches

Reliable measurement of β-defensins is essential for evaluating their potential as early biomarkers in prediabetes rather than solely as indicators of established disease. When studying HBD protein levels, most researchers typically rely on enzyme-linked immunosorbent assay (ELISA) methods. The commercial kits can differ significantly in their sensitivity and specificity. Some are specifically designed for total defensin detection (30), while others focus on isoforms like HBD-1, HBD-2, or HBD-3 (31). These methodological differences can lead to inconsistencies in results from various studies. Moreover, the type of sample used plays a significant role in determining the effectiveness of detection levels (32). To generate comparable data across studies, especially when evaluating HBDs as potential biomarkers for diabetes, it’s crucial to standardize ELISA protocols.

A 2023 study by JG Routsias and his team focused on measuring the serum concentration of HBD2. They utilized a commercial ELISA development kit from Peprotech, Inc. (London, UK), which operates in a sandwich ELISA format. To ensure the patients’ blood serum were suitable for the assay, they were diluted to 1:5 ratio, allowing them to fall within the dynamic range of 16–2,000 pg/mL. With a cutoff value of 655 pg/mL. The test showed a sensitivity of 78.0% and a specificity of 93.2% in distinguishing between infectious and non-infectious inflammation (30). Another study from the same year measured salivary HBD-1 using a Peprotech ELISA kit (Cat. No. 900-M202) at a 1:30 dilution, adhering to the sandwich ELISA principle, which has a detection limit of 4 pg/mL. They quantified salivary HBD-2 using the Peprotech kit (Cat. No. 900-M172) from undiluted samples, with a detection limit of 16 pg/mL. Salivary HBD-3 was also analyzed using the Peprotech kit (Cat. No. 900-M210) from undiluted samples, with a detection limit of 62 pg/mL (33). In 2021, Fusco et al., measured the levels of HBD-2 and -3 in cell-free supernatants from transfected Caco-2 intestinal epithelial cells by using ELISA kits from Elabscience for HBD-2 and Abcam for HBD-3. They discovered that HBD-2 concentrations were around 200 pg/mL in Caco-2/HBD-2 cells, while HBD-3 levels were around 90 pg/mL in Caco-2/HBD-3 cells (34). A 2022 study studied saliva from patients diagnosed with type 2 diabetes and chronic periodontitis. The researchers quantified HBD-2 using an ELISA kit from Jiangxi Albain Biotechnology (Cat. IB-E20311). They found that those with poor glycemic control and moderate to severe chronic periodontitis had HBD-2 levels of about 4.2 ng/mL. Meanwhile, the good control group with mild chronic periodontitis showed levels of roughly 1.0 ng/mL, indicating that the first group had around four times higher levels (6). Most studies measure HBDs using sandwich ELISAs; however, considerable variability must be considered. The differences in kit formats, such as development kits versus ready-to-use ones, and the specificity for isoforms (HBD-1, HBD-2, HBD-3), along with whether serum or saliva is used as the matrix, can lead to notable variations in research outcomes. A key limitation is the lack of harmonization across studies; different ELISA kits often give results that can’t be directly compared. This inconsistency makes it difficult to determine actual HBD levels and underscores the need for standardized calibration and reporting. Understanding the strengths and limitations of these assays is crucial, as they significantly influence the evidence linking HBD expression to diabetes in both type 1 and type 2. A comparative overview of ELISA platforms, sample matrices, detection limits, and reported defensin concentrations across key clinical and experimental studies is summarized in **Table 1**.

**Table 1:** ELISA-based quantification of human β-defensins across serum, saliva, and epithelial cell systems.

Study (Year)	Sample type	HBD isoform	ELISA kit (Company)	Sample preparation	Detection range/Limit	Cut-off	Key findings
Routsias et al., 2023 (30)	Serum	HBD-2	PeproTech (London, UK) – Development Kit (sandwich ELISA)	1:5 dilution	16–2,000 pg/mL	655 pg/mL	Serum HBD-2 distinguished infectious from non-infectious inflammation with 78.0% sensitivity and 93.2% specificity
Gursoy et al., 2023 (33)	Saliva	HBD-1	PeproTech (Cat. No. 900-M202)	1:30 dilution	4 pg/mL (detection limit)	Not defined	Quantified basal salivary HBD-1 levels
Gursoy et al., 2023 (33)	Saliva	HBD-2	PeproTech (Cat. No. 900-M172)	Undiluted	16 pg/mL (detection limit)	Not defined	Measured salivary HBD-2 concentrations
Gursoy et al., 2023 (33)	Saliva	HBD-3	PeproTech (Cat. No. 900-M210)	Undiluted	62 pg/mL (detection limit)	Not defined	Measured salivary HBD-3 concentrations
Fusco et al., 2021 (34)	Caco-2 cell culture supernatant	HBD-2	Elabscience (ELISA kit)	Cell-free supernatant	Not reported	Not applicable	HBD-2–transfected Caco-2 cells secreted ≈200 pg/mL HBD-2 into the culture medium
Fusco et al., 2021 (34)	Caco-2 cell culture supernatant	HBD-3	Abcam (ELISA kit)	Cell-free supernatant	Not reported	Not applicable	HBD-3–transfected Caco-2 cells secreted ≈90 pg/mL HBD-3 into the culture medium
Xiao et al., 2022 (6)	Saliva (T2DM patients with periodontitis)	HBD-2	Jiangxi Albain Biotechnology (Cat. No. IB-E20311)	Not reported	Not reported	Not defined	Salivary HBD-2 was markedly elevated in poorly controlled T2DM with severe periodontitis (~4.2 ng/mL) compared with well-controlled diabetes (~1.0 ng/mL)

This methodological heterogeneity represents a major barrier to translating β-defensin measurements into clinically meaningful biomarkers for prediabetes. As summarized in **Table 1**, substantial methodological heterogeneity exists in HBD quantification across studies, reflecting differences in sample matrices, ELISA platforms, antibody specificity, and assay sensitivity. These methodological differences likely contribute to the wide range of reported HBD concentrations (pg/mL to ng/mL) and to the inconsistent directionality of defensin changes reported across diabetes studies (summarized in **Table 1**). Importantly, salivary HBD-2 shows the most consistent elevation in poorly controlled T2DM with periodontitis (6), while pancreatic and epithelial HBD-1 and HBD-3 are reduced in T1DM and hyperglycemic states (7). This pattern supports the view that HBDs reflect tissue-specific immune and metabolic stress rather than uniform systemic changes, reinforcing the need for standardized assays before HBDs can be validated as metabolic biomarkers.

## 4 Lessons from established diabetes: implications for prediabetes

Evidence from established diabetes provides mechanistic insight into immune–metabolic alterations that are likely initiated earlier, during prediabetes (35). Across both type 1 and type 2 diabetes, consistent disturbances in β-defensin biology point to impaired epithelial immunity, altered inflammatory signaling, and disrupted host–microbe interactions rather than nonspecific consequences of hyperglycemia (4, 36).

### 4.1 Pancreatic defensin loss in T1DM

Human pancreatic biopsies from individuals with type 1 diabetes show a marked reduction in HBD-1 and HBD-3 expression in both endocrine and exocrine compartments, independent of local inflammatory infiltration. This pattern suggests an intrinsic failure of innate immune defense within the pancreas rather than secondary suppression by insulitis (7). Loss of defensins at this site may weaken antimicrobial protection, amplify inflammatory stress, and increase β-cell vulnerability, supporting a role for defensin deficiency in sustaining immune-mediated β-cell dysfunction.

### 4.2 Salivary HBD alterations in T2DM

In type 2 diabetes, the most reproducible changes in β-defensins are observed in the oral compartment. Salivary HBD-2 levels are significantly elevated in individuals with poor glycemic control and correlate with HbA1c and inflammatory burden (6). These alterations likely represent a compensatory epithelial response to combined metabolic and inflammatory stress rather than a direct effect of diabetes alone. Importantly, the association between salivary HBD-2 and glycemic control suggests that defensin dysregulation tracks metabolic deterioration and may emerge before overt diabetes.

### 4.3 Mechanistic implications

Experimental data supports a functional role for β-defensins in modulating inflammatory and metabolic pathways relevant to insulin resistance. HBD-3 suppresses TLR4–MyD88/TRIF–NF-κB signaling in human macrophages, a pathway strongly implicated in endotoxin-driven inflammation and systemic insulin resistance (23). In diet-induced obese mice, oral administration of HBD-2 improves glucose tolerance, reduces hepatic steatosis, and strengthens intestinal barrier integrity, implicating a defensin–microbiota–endotoxemia axis in metabolic regulation (5). Complementary animal studies demonstrate that insulin treatment partially restores β-defensin expression and Toll-like receptor balance under hyperglycemic conditions, highlighting bidirectional crosstalk between metabolic control and innate immunity (37).

### 4.4 Relevance to prediabetes

Collectively, these findings indicate that β-defensin alterations in diabetes are tissue-specific, mechanistically meaningful, and not merely epiphenomena of advanced disease. Pancreatic defensin loss points to early vulnerability of β-cell–associated innate immunity, while salivary HBD-2 elevation mirrors escalating metabolic and inflammatory stress (6, 7). The causal improvements observed in animal models further support the concept that defensin dysregulation may precede and contribute to insulin resistance rather than simply reflect it. These lessons from established diabetes therefore provide a strong rationale for investigating β-defensins as early immune–metabolic markers during the prediabetic stage.

## 5 Pre-diabetes to diabetes transition

The transition from pre-diabetes to diabetes reflects early immune–metabolic dysregulation rather than glycemia alone. While insulin resistance, low-grade inflammation, and progressive β-cell stress are well-established drivers of this transition (38–44), emerging evidence suggests that human β-defensins (HBDs) may participate in these early events by modulating epithelial immunity, inflammatory tone, and gut barrier integrity (7, 23). However, β-defensins have rarely been examined longitudinally during pre-diabetes, and their contribution to disease progression remains poorly defined.

Insulin resistance (IR) is one of the initial and most significant changes observed in pre-diabetes. The primary tissues involved in glucose disposal, such as skeletal muscle and liver, become less sensitive to insulin. Consequently, this results in impaired glucose uptake and storage (45). A recent study with a prospective cohort showed that rising HOMA-IR levels are a strong predictor of the transition from pre-diabetes to T2DM. This finding emphasizes that decreased insulin sensitivity can take place years before fasting glucose or HbA1c levels reach the diabetic range (46). Whether early insulin resistance is accompanied by parallel changes in β-defensin expression in metabolic tissues remains largely unexplored.

In addition to IR, chronic low-grade inflammation is critical. For those who are pre-diabetic, their fat tissue often releases pro-inflammatory cytokines such as TNF-α and IL-6 (47). Some factors can disrupt insulin receptor signaling and worsen metabolic health. A large-scale study that monitored middle-aged women over several years found that those with higher levels of IL-6 in their blood at the start were more likely to develop T2DM, even when accounting for other risk factors like BMI or family history (48).

There’s increasing evidence that the gut microbiome plays a crucial role in the development of pre-diabetes. Studies using multi-omics approaches have found that lower microbial diversity and a reduction in short-chain fatty acid (SCFA)-producing bacteria are associated with impaired insulin sensitivity and increased inflammation. For example, a 2023 metabolomic study highlighted that enhanced microbial metabolism of carbohydrates into monosaccharides was associated with higher pro-inflammatory cytokine levels and worsened insulin resistance (49). In a 2025 dietary intervention study, researchers discovered that enriching butyrate-producing species significantly improved insulin sensitivity in individuals who have pre-diabetes (50). These findings suggest that pre-diabetes is far more than just a case of slightly elevated blood sugar. It’s a complex issue where the body develops insulin resistance, fat tissue releases inflammatory signals like IL-6 and TNF-α, and the gut microbiome changes in ways that can hinder metabolism. All these factors create pressure on the pancreatic β-cells, resulting in the development of diabetes.

### 5.1 Gut microbiome and defensins

**Figure 1:**
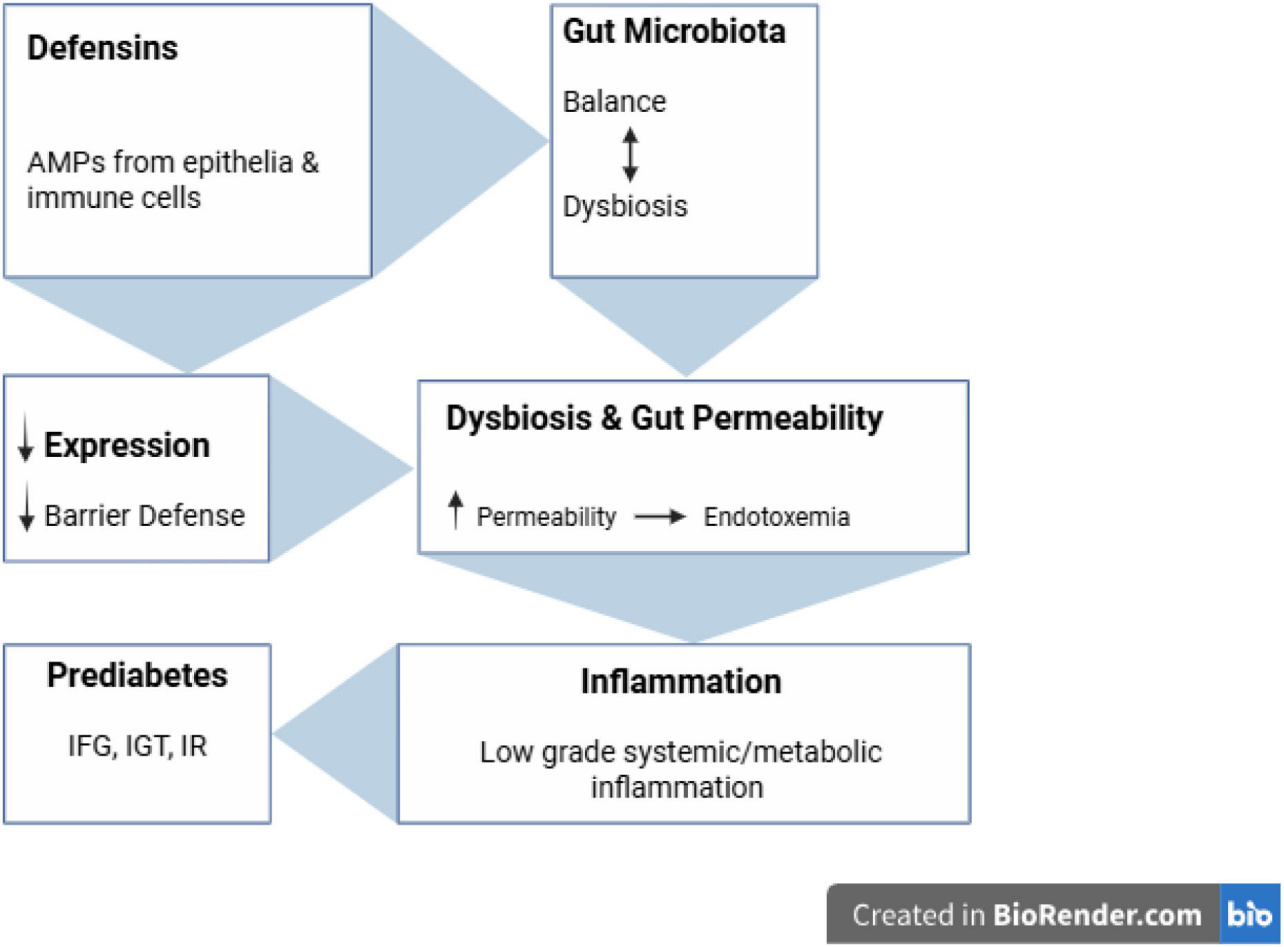
Defensins, gut microbiota, and the link to prediabetes. Defensins sustain gut barrier integrity, while their reduction disrupts microbiota balance, increases permeability and endotoxemia, and promotes systemic inflammation leading to insulin resistance and prediabetic states (IFG, IGT, IR).

Paneth cell α-defensins maintain epithelial barrier integrity and shape microbiota composition; reduced defensin activity drives dysbiosis, metabolic endotoxemia, and low-grade systemic inflammation that promotes insulin resistance (51). In animal models, vitamin D deficiency, when combined with a high-fat diet, suppresses α-defensins, increases gut permeability, and precipitates insulin resistance and steatosis. Conversely, oral administration of human α-defensin (DEFA5) restores gut balance and improves metabolic outcomes (52). Gut microbiome alterations in prediabetes include loss of butyrate-producing bacteria and increased gut permeability, which may interact with defensin pathways (53). The processes are described briefly and clearly in **Figure 1**.

### 5.2 Hypothesized role of HBDs in the pre-diabetes to diabetes transition

Defensins, as immune effectors, may be involved in this inflammatory milieu. While direct human studies on defensins in prediabetes are limited, their role in modulating inflammation and gut barrier integrity suggests a mechanistic link. HBDs may play a crucial role in the journey from pre-diabetes to full diabetes by influencing inflammation and immune regulation. These antimicrobial peptides are known for their ability to attract immune cells, including those that secrete IL-6 and TNF-α. This could worsen the chronic low-grade inflammation that’s often seen in pre-diabetes. Additionally, emerging research suggests that changes in HBD levels in metabolic tissues or the bloodstream might reflect early signs of conflicts in the body’s defense and metabolic regulation. A study found that patients with T2DM and periodontitis had elevated levels of salivary HBD-2 and HBD-3 compared to healthy controls. This suggests that defensins may be increasing in response to both inflammatory and metabolic stress (54, 55). Furthermore, certain genetic variations in the DEFB1 gene have been associated with an increased risk of complications from T2DM, suggesting a potential genetic impact on the activity of defensins (18).

### 5.3 Potential predictive value

Studies have shown that HBDs could be promising early indicators of the progression from pre-diabetes to diabetes. Their presence in saliva, plasma, or tissue samples enables a non-invasive method for tracking inflammatory and metabolic shifts. While we don’t have extensive long-term research on pre-diabetic individuals yet, confirming HBD markers could pave the way for monitoring their levels. This could be done either on their own or in conjunction with other valuations like HOMA-IR or β-cell function, making it easier to pinpoint those who are likely to develop T2DM (6, 20, 56).

These processes highlight pre-diabetes as a dynamic state of immune-metabolic imbalance, paving the way to explore how HBDs may act as predictive signals or modulators during this stage.

## 6 Downstream consequences of early immune–metabolic dysregulation

Many diabetes-related complications originate from immune and metabolic disturbances that are already present during prediabetes, rather than arising solely from prolonged hyperglycemia. Large population-based studies consistently demonstrate increased cardiovascular, renal, neurological, and infectious risk in individuals with diabetes; however, these outcomes likely reflect early immune–metabolic vulnerability that begins years before the diagnosis of overt disease (57–61).

Cardiovascular complications provide a clear example of this continuum. In a nationwide Swedish cohort of over 679,000 individuals with type 2 diabetes, the risk of heart failure remained approximately 50% higher than in non-diabetic controls, even when traditional cardiovascular risk factors were well controlled (57). From a prediabetes perspective, these findings suggest that vascular vulnerability is established early and may be driven by chronic low-grade inflammation and impaired innate immune regulation. Given the role of β-defensins in epithelial and endothelial defense, early defensin imbalance may contribute to the inflammatory milieu that predisposes to later cardiovascular disease.

Infectious susceptibility further highlights early immune dysfunction along the dysglycemic spectrum. Large cohort studies have shown that both insulin resistance and abnormal glucose levels are associated with increased infection-related mortality, independent of overt diabetes status (60). Similarly, hospitalization for pneumonia in individuals with type 2 diabetes is linked to elevated short- and long-term cardiovascular mortality (61). These observations point to compromised innate immune defenses that are likely to emerge during prediabetes. Because β-defensins are central mediators of mucosal and epithelial immunity, early dysregulation of defensin expression may contribute to infection vulnerability rather than merely reflecting established metabolic disease.

Experimental and clinical data further implicate β-defensins in vascular and tissue-specific complications. Reduced expression of DEFB1 in cardiac tissue has been associated with increased coronary artery disease risk, suggesting loss of local innate protection (62). Conversely, abnormal release of HBD-3 from activated platelets has been shown to induce endothelial dysfunction and vascular injury, indicating that dysregulated defensin activity can shift from protective to pathogenic (63). Notably, *in vitro* studies also demonstrate that HBD-3 can suppress TNF-α–mediated inflammation and preserve endothelial integrity under controlled conditions (64), underscoring the context-dependent effects of defensins on vascular homeostasis.

Taken together, these downstream complications reinforce the concept that immune–metabolic dysregulation begins during prediabetes and that β-defensin imbalance may act as an early contributor to vascular, infectious, and tissue-specific vulnerability rather than solely representing a consequence of advanced diabetes.

## 7 HBDs as diagnostic, prognostic, and therapeutic biomarkers

Clinical studies have measured HBDs in saliva, blood, and wound samples, showing disease-related changes. As discussed in Section 4, salivary HBD alterations in T2DM reflect epithelial responses to combined metabolic and inflammatory stress. From a biomarker perspective, this supports salivary HBD-2 as a non-invasive indicator of disease burden rather than a diabetes-specific marker.

Patient studies already highlight HBDs as potential diagnostic, prognostic, and therapeutic biomarkers. In one hospital cohort of 114 patients, serum HBD-2 distinguished infectious from non-infectious inflammation with high accuracy (AUC = 0.90), outperforming traditional markers like C-reactive protein (CRP) and procalcitonin (PCT) (30). In preclinical work, alginate hydrogels releasing HBD-2 accelerated wound healing and reduced MRSA burden in diabetic mice, pointing to defensins as both treatment-response biomarkers and candidate therapies that now need to be tested in human trials (65). Earlier preclinical work by Hirsch et al. demonstrated that topical application of HBD-3 accelerated wound healing in infected diabetic wounds, underscoring the therapeutic potential of defensins beyond their antimicrobial effects (66). A synthetic θ-defensin (RTD-1) improved insulin action and normalized glucose and free fatty acids in obese rats, suggesting therapeutic potential for insulin resistance and type 2 diabetes (67). Recent computational work has further strengthened this view, showing that HBD-2 may act as more than an antimicrobial peptide. Molecular modeling suggests it carries dual functions, combining immune defense with wound-healing capacity, which is especially relevant in the diabetic setting. These findings open the door for developing HBD-2-based therapies aimed at accelerating tissue repair in diabetes, aligning computational insights with the clinical need for innovative interventions (68).

To provide a comprehensive overview, **Table 2** summarizes original studies of defensins across human cohorts, *in vitro* cell models, and *in vivo* animal studies, highlighting their roles in diabetes, prediabetes, and metabolic dysfunction. Still, most biomarker studies remain cross-sectional, so we cannot determine whether alterations in defensin lead to metabolic deterioration or reflect it. Yet, to fully grasp their predictive value, we need to examine how HBDs behave specifically in pre-diabetes, across their different subgroups.

**Table 2:** Original studies on defensins in prediabetes and diabetes.

HBD isoform(s)	Group	Study type	Sample/System	Method	Key findings	Authors
HBD-1 (DEFB1)	Type 2 Diabetes + periodontitis	Human	Gingival tissue	Gene expression + SNPs	DEFB1 polymorphisms linked to periodontitis in T2D; ↑ local HBD-1 expression.	(2023) Subbiah et al.
HBD-1 (DEFB1)	Type 2 Diabetes	Human	Blood (genetic)	Genotyping	DEFB1 variants associated with diabetic kidney disease progression.	(2022) Ochoa-Ramírez et al.
HBD-2	Obese & overweight adults (prediabetes/IR risk)	Human	Serum	ELISA	Circulating HBD-2 correlated with BMI and inflammatory markers; mirrors low-grade inflammation.	(2025) Koufakis et al.
HBD-2	Diabetic wound model	*In vivo*	Alginate hydrogel with HBD-2	Preclinical wound assay	Accelerated wound healing, reduced MRSA burden; therapeutic potential.	(2025) Da Silva et al.
HBD-2	Obese mice	*In vivo*	Oral defensins	Animal intervention	Oral HBD-2 improved glucose tolerance, ↓ hepatic steatosis, ↑ gut barrier integrity.	(2023) Rosa et al.
HBD-2	Type 2 Diabetes + periodontitis	Human	Saliva	ELISA	Poor glycemic control + severe periodontitis → ~4× higher salivary HBD-2.	(2022) Xiao et al.
HBD-3	Type 1 Diabetes (children/adolescents)	Human	Saliva	ELISA	Salivary HBD-3 reduced regardless of gingival inflammation; early AMP imbalance.	(2022) Yılmaz et al.
HBD-3	High-glucose exposure	*In vitro*	Human keratinocytes	Cell culture/p38MAPK	High glucose reduced HBD-3 via inhibition of p38MAPK signaling.	(2011) Lan et al.
HBD-3	Human macrophage assays	*In vitro*	Macrophages	Pathway studies	HBD-3 attenuated MyD88/TRIF–NF-κB inflammatory signaling → potential to reduce IR.	(2011) Semple et al.
HBD-1, HBD-3	Type 1 Diabetes (adults)	Human	Pancreatic biopsies	Tissue immunostaining/gene expression	Reduced HBD-1 and HBD-3 in endocrine & exocrine pancreas, independent of inflammation.	(2024) Tegehall et al.
HBD-1, HBD-2, HBD-3	With/without T2D + prediabetes	Human	Saliva	ELISA	Salivary HBD-2/3 decreased with rising HbA1c; link to glycemia before overt diabetes.	(2020) Yılmaz et al.
HBD-2, HBD-3	Intestinal epithelial barrier model	*In vitro*	Caco-2 supernatants	ELISA	HBD-2/3 enhanced barrier integrity under Candida stress.	(2021) Fusco et al.
α-defensins (Paneth)	Obese mice + vitamin D–deficient	*In vivo*	Gut microbiota model	Animal model	Induction of α-defensins restored microbiota, improved steatosis and metabolic outcomes.	(2016) Su et al.
θ-defensin (RTD-1)	Obese rats	*In vivo*	Systemic	Metabolic assays	Improved insulin action, normalized glucose & FFAs.	(2015) Oh et al.

## 8 Role of HBDs in pre-diabetes

### 8.1 Subgroups of pre-diabetes and their risk

Direct human evidence linking β-defensins to prediabetes remains strikingly limited, representing a major gap in current immunometabolic research. Prediabetes is not just one condition; it’s a collection of different subgroups that have their own unique biological characteristics and varying chances of developing into diabetes. For instance, individuals with impaired fasting glucose (IFG) often experience their liver producing too much sugar during the night (69). In the case of people with impaired glucose tolerance (IGT), where their muscles aren’t as effective at absorbing sugar post-meal, roughly 4% progress to diabetes each year (70). The combined IFG+IGT group raises the most alarm, with nearly 15–20% of people progressing to diabetes each year, placing it in the highest-risk category. Another cohort is identified by HbA1c values ranging from 5.7% to 6.4%, indicating sustained elevations in blood glucose levels. This group is subject to an annual risk estimated at approximately 5–7% (55). In the United States, it’s estimated that one in five adolescents and one in four young adults are living with prediabetes, with the numbers being exceptionally high among males and those who are overweight. This group of young people faces a troubling cardiometabolic risk profile, which increases their chances of developing type 2 diabetes and cardiovascular issues. Specifically, 11.1% of adolescents and 15.8% of young adults experience IFG, while around 4% of adolescents and 6% of young adults have IGT. Additionally, both demographics show a marked reduction in insulin sensitivity compared to their peers who maintain normal glucose levels (P < 0.05) (71). **Figure 1** illustrates how alterations in defensins may disrupt intestinal homeostasis, compromise the epithelial barrier, and promote inflammatory processes that contribute to the progression from prediabetes to diabetes. Understanding these categories is crucial since each one carries unique risks for diabetes, cardiovascular issues, and other complications. This highlights the need for prevention strategies, whether they involve lifestyle adjustments or targeted therapies, to be specifically tailored to each subgroup. **Figure 1** illustrates how defensins interact with the gut microbiota and barrier integrity, linking dysbiosis, inflammation, and insulin resistance in prediabetes.

### 8.2 Experimental evidence (*in vitro* & animal)

Initial evidence suggests that HBDs may behave differently across the subtypes of pre-diabetes. In hyperglycemic conditions, the behavior of HBD-2 and -3 expressions stands apart from HBD-1. It turns out that keratinocytes exposed to high glucose levels show a reduction in the expression of HBD-2 and -3 (22). In parallel, hyperglycemia-associated dicarbonyls (methylglyoxal/glyoxal) modifies hBD-2 and blunt its activity, indicating that early metabolic stress may degrade defensin function even before overt diabetes (21). Experimental findings in mice suggest that treatment with HBD-2 not only improves glucose metabolism but also contributes to the repair of the gut barrier, which is often damaged in metabolic diseases (5). A study from 2019 found that HBDs play a significant role in fighting insulin resistance. They do this by inhibiting the production of glucocorticoids, which helps reduce chronic inflammation. Additionally, HBDs activate the Toll-like receptor signaling pathway, encouraging cell migration, proliferation, angiogenesis, and the stabilization of fibroblasts and keratinocytes (72). Animal studies add another layer to this link; in diabetic mice, restraint stress raised endogenous glucocorticoids and caused a marked drop in esophageal β-defensin-3 expression, an effect reproduced by exogenous glucocorticoids, suggesting that stress hormones may directly suppress mucosal defensin defenses (73). HBDs may not only indicate inflammation; they could also provide information about early immune changes associated with diabetes.

### 8.3 Early human evidence

**Figure 2:**
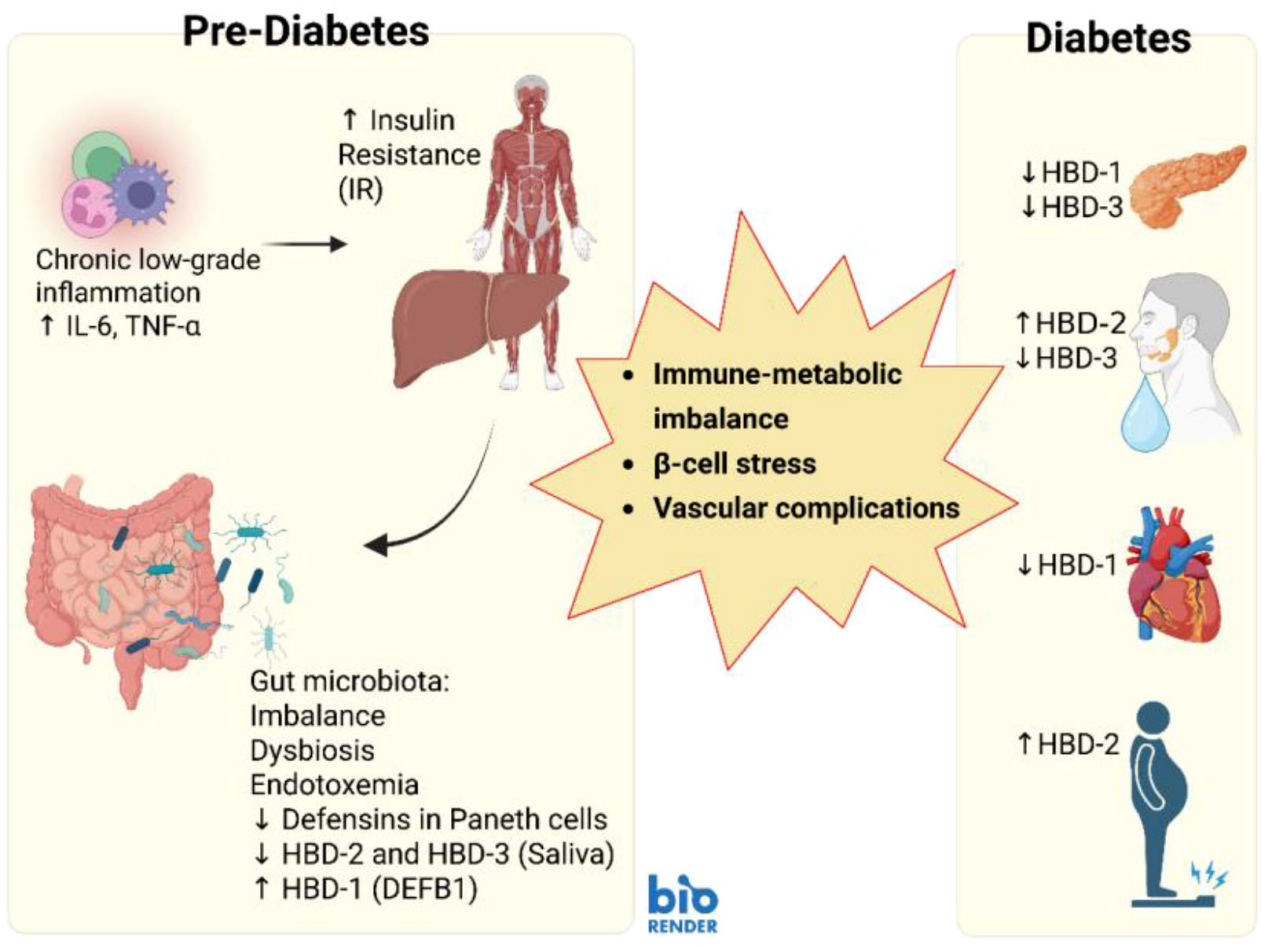
Expression of β-Defensins in Pre-diabetes and Diabetes. This figure illustrates the differences in β-defensin behavior between pre-diabetes and diabetes. In the pre-diabetic stage, changes are still mild; defensins begin to shift in response to early immune stress, insulin resistance, and gut barrier alterations. Once diabetes develops, the picture becomes clearer and more intense: HBD-1 and HBD-3 drop in the pancreas, salivary HBD-2 and HBD-3 fluctuate with blood sugar control, and abnormal defensin release may add to vascular and metabolic problems. Taken together, these differences underline that HBDs are not static but adapt to disease stage, making them promising markers for identifying and tracking metabolic health.

Although there isn’t extensive direct research specifically linking prediabetes and β-defensin, a study in 2022 suggested a connection between high glucose levels, which are characteristic of prediabetes, and dysregulation in expression of HBDs. Specifically, high glucose levels may decrease certain β-defensins, such as DEFB4A (HBD-2), potentially compromising gut barrier integrity and contributing to metabolic dysregulation in human uroepithelial cells. At the same time, DEFB103A (HBD-3) remained unchanged (74). When human primary epithelial cells were exposed to high glucose (30 mM) or very low insulin levels (<5 µg/mL), their production of HBD-2 and -3 dropped noticeably (Williams, unpublished) (75). In support of these findings, another study found that even in those without diabetes, a rise in HbA1c levels was often accompanied by a decrease in salivary HBD-2 and HBD-3 (56). In an *in vitro* study, Barnea et al. demonstrated that raising intracellular glucose levels in human embryonic kidney cells led to an increase in HBD-1 expression (76). In Honkanen et al., from the fecal samples of children who already presented β-cell autoimmunity and later developed type 1 diabetes had higher levels of HBD-2 than those who didn’t (77). In patients with insulin resistance, HBD-1 exhibited strong mRNA expressions in the esophagus and antrum, but its expression was significantly lower in the corpus and duodenum. In contrast, HBD-4 mRNA was consistently detected across all groups throughout the entire gastrointestinal tract (78). (**Figure 2**) summarizes these observations by comparing β-defensin expression in pre-diabetes and diabetes, highlighting stage-specific changes across tissues and fluids.

### 8.4 Summary

Currently, there is limited direct human evidence linking β-defensins to prediabetes, and the existing research remains in the preliminary stages. To strengthen the evidence base, further research should systematically assess β-defensins across well-defined prediabetes phenotypes (IFG/IGT/HbA1c 5.7–6.4%) in conjunction with HOMA-IR measurements. To visualize these findings, **Figure 2** compares β-defensin expression patterns observed in pre-diabetes and diabetes, emphasizing stage-specific alterations across tissues and fluids. Most of the current evidence comes from *in vitro* studies, while the few available human studies are generally small and lack sufficient statistical power. Until larger, well-characterized cohorts across IFG, IGT, and HbA1c subgroups are studied, the role of defensins as early predictors should be considered preliminary. These early signs suggest that defensins may link immune stress in pre-diabetes to metabolic decline, supported by their growing associations with lipid metabolism. Together, as shown in **Figures 1** and **2**, defensins shift in response to metabolic stress and disease stage, underscoring their potential as early markers.

## 9 Link between HBDs and dyslipidemia

Dyslipidemia represents a key metabolic stressor that intersects with innate immune activation. Emerging evidence suggests that β-defensins may act as immune sensors of lipid-driven inflammation, linking altered lipid metabolism to chronic inflammatory signaling. Dyslipidemia isn’t just a minor detail in metabolic syndrome; it plays a vital role and is a significant risk factor. To put it simply, this means that people with metabolic syndrome often have an unhealthy lipid profile, which includes high triglycerides and VLDL, small dense LDL (which is particularly dangerous), and low levels of good HDL cholesterol. This imbalance directly contributes to atherosclerosis and vascular issues, even affecting the heart muscle cells at a cellular level (79). In a large cohort of over 92,000 adults, researchers found that atherogenic dyslipidemia, marked by high triglycerides and low HDL, carried a far more dangerous cardiovascular risk profile than having either problem alone. It was also more likely to cluster with other risks like high blood pressure, diabetes, obesity, and metabolic syndrome, making it a particularly concerning form of dyslipidemia (80).

These lipid changes are directly tied to inflammation in the blood vessels, the instability of plaques, and the worsening of fatty liver disease. This explains why dyslipidemia isn’t merely a byproduct; it’s a fundamental contributor to the complications associated with metabolic syndrome (81). It’s been recognized for a long time that macrophages in atherosclerotic plaques produce different inflammatory cytokines and chemokines when they take up too many lipoproteins and become foam cells (82). A study showed that lipid droplets act like command centers inside macrophages, teaming up with mitochondria, the ER, and peroxisomes to handle fats. By working together, these organelle clusters control how fatty acids are stored or released, directly shaping the cell’s ability to produce inflammatory signals (83). It appears that the level of lipids within cells is linked to how we react to inflammation. When macrophages get activated by TLRs, they lead to an increase in cholesterol and triglycerides. This occurs as they promote the uptake of lipoproteins and free fatty acids, which enhances the process of lipogenesis (84, 85). The results demonstrate that metabolism plays an active and regulatory role in shaping immune system responses, rather than functioning solely as a background process.

It’s notable to consider that the beta defensins might also be involved in how our bodies process fats. A 2025 study by Theocharis Koufakis et al. included 81 infection-free adults, grouped by body weight into normal, overweight, and obese categories. Researchers measured HBD-2, along with key inflammation markers, and found that higher HBD-2 levels were associated with more systemic inflammation in obese individuals. Although the study did not confirm cause and effect, it highlighted a possible role for HBD-2 as an indicator of inflammation in obesity (86). In a study involving male C57BL/6J mice on a Western-style diet rich in fats and sugars, researchers administered HBD-2 orally for six weeks. Afterward, they evaluated the liver fat content, glucose tolerance, and markers indicating the integrity of the gut barrier. The results strongly suggest that treatment with β-defensins can enhance liver fat levels, improve glucose metabolism, and support gut barrier function (5). Annika Linde’s group demonstrated that a high-fat diet upregulates cardiac rat β-defensin genes, with eight of ten increasing, with rBD10, rBD11, and rBD33 being the most pronounced. This suggests excess dietary fat can activate cardiac β-defensins, promote monocyte migration, and amplify innate-immune–driven inflammatory stress in the heart (87). It looks like β-defensins are bridging the gap between fat metabolism and our innate immunity. They indicate inflammation in obesity, enhance liver fat management in mice fed a high-fat diet, and their expression goes up with increased fat consumption. Together, these findings suggest that defensins serve as integrators of metabolic and immune pathways, laying the groundwork for a broader understanding of their role and potential future applications.

## 10 Conclusion and future perspectives

Based on the available evidence, β-defensins most reasonably function as early immune–metabolic modulators whose dysregulation may precede and contribute to the progression from prediabetes to diabetes. However, recent research has unveiled their significant involvement in the metabolic and inflammatory processes that contribute to diabetes and pre-diabetes. Genetic studies back this up, showing that specific variants of the DEFB1 gene (like rs11362 and rs1799946) are more common in people with diabetes and its related complications. This connection suggests that defensins play a role in chronic low-grade inflammation and insulin resistance (18, 20, 25). Clinical studies showed that HBD levels can vary depending on glycemic control. For instance, in individuals with poorly managed type 2 diabetes who also had periodontitis, there’s a significant increase in salivary HBD-2 levels (6, 33), whereas in type 1 diabetes, pancreatic tissue exhibited a notable decrease in HBD-1 and HBD-3, indicating a compromised innate defense system (7). Even prior to the onset of diabetes, keratinocytes that were exposed to elevated glucose levels tend to lose their expression of HBD-2 and HBD-3 (22), suggesting that defensin imbalance is an early feature of metabolic stress.

Mechanistic research provides a basis for understanding these clinical patterns. Experimental models have demonstrated that HBD-3 can attenuate TLR4–NF-κB inflammatory signaling (23). On the other hand, oral delivery of HBD-2 in obese mice improves glucose tolerance, reduces hepatic fat accumulation, and strengthens gut barrier function (5). These findings signify that HBDs are not just markers of disease but may actively form the metabolic and immune pathways, hypothetically limit insulin resistance, and support tissue repair (72).

From a translational perspective, HBDs hold promises as both biomarkers and therapeutic tools. Their detectable levels in saliva, serum, and tissues make them appealing for non-invasive tracking of metabolic stress and systemic inflammation. Serum HBD-2 has already demonstrated high diagnostic accuracy in distinguishing between infectious and non-infectious inflammation, outperforming CRP and PCT (30). Similarly, salivary HBD-2 tracks worsening glycemic control in type 2 diabetes (6), while reduced salivary HBD-3 in youth with type 1 diabetes signals early immune imbalance (88). Beyond just diagnostics, there’s exciting potential in using alginate hydrogels infused with HBD-2 to accelerate wound healing and reduce the MRSA burden in diabetic mice. This highlights how defensins might not only act as indicators of treatment response but also serve as innovative therapeutic agents (65). Taken together, HBDs appear to serve a double role in diabetes and pre-diabetes: as early cautionary signals of disease progression and as promising targets for innovative therapies. Looking ahead, future work should examine how HBD patterns vary by age, sex, ethnicity, obesity, and genetics to determine if these peptides could help guide more tailored, precision-based care. Their integration into clinical practice, if validated in larger trials, may open new avenues for early intervention and precision care in metabolic disease (30, 54–56, 65, 88). From a translational standpoint, future studies should validate HBDs in large, multicenter cohorts and assess their added value beyond traditional markers such as HbA1c, HOMA-IR, and OGTT. Incorporating HBD testing into existing risk models could improve the precision of identifying individuals at high risk for progression. Equally important, cost-effectiveness analyses are needed to determine whether salivary or blood-based HBD assays provide practical advantages over currently used biomarkers, especially in routine clinical settings.

Despite the growing body of evidence linking β-defensins to metabolic and inflammatory dysregulation, significant gaps remain that currently limit their translation into clinical practice. To advance the field, future research must adopt a clear, stepwise approach. First, prospective longitudinal human cohort studies spanning the full spectrum of prediabetes (IFG, IGT, and HbA1c-defined states) are essential to determine whether alterations in β-defensin profiles precede the onset of overt diabetes rather than merely reflecting established disease. Building on this foundation, standardized and harmonized methodologies for β-defensin measurement, particularly in saliva and serum, are required to ensure reproducibility and enable meaningful comparison across studies. Once robust measurement frameworks are established, integrating β-defensin profiling with established metabolic markers such as HbA1c, OGTT, and HOMA-IR may improve risk stratification and mechanistic insight. Ultimately, interventional and mechanistic studies in high-risk prediabetic populations will be necessary to clarify whether modulation of defensin pathways can meaningfully alter immune–metabolic trajectories and delay or prevent progression to diabetes.
